# Targeting the Interaction Between Spike Protein and Nucleocapsid Protein for Suppression and Detection of Human Coronavirus OC43

**DOI:** 10.3389/fimmu.2022.835333

**Published:** 2022-03-10

**Authors:** Jinsoo Kim, Minyoung Kim, Dongbum Kim, Sangkyu Park, Mijeong Kang, Kyeongbin Baek, Jun-Kyu Choi, Sony Maharjan, Madhav Akauliya, Younghee Lee, Hyung-Joo Kwon

**Affiliations:** ^1^ Department of Microbiology, College of Medicine, Hallym University, Chuncheon, South Korea; ^2^ Institute of Medical Science, College of Medicine, Hallym University, Chuncheon, South Korea; ^3^ Department of Biochemistry, College of Natural Sciences, Chungbuk National University, Cheongju, South Korea

**Keywords:** HCoV-OC43, spike protein, nucleocapsid protein, anti-HCoV-OC43 N antibody, bait and prey, virus detection, ELISA

## Abstract

Human coronavirus OC43 (HCoV-OC43) is the coronavirus most associated with “common colds”, infections of the upper respiratory tract. Previously, we reported that direct interactions of nucleocapsid (N) protein and C-terminal domain of Spike protein (Spike CD) are essential for replication of SARS-CoV-2 and MERS-CoV. Thus, we developed a novel ELISA-based strategy targeting these specific interactions to detect SARS-CoV-2 and MERS-CoV. Here, we investigated whether the same principles apply to HCoV-OC43. We discovered that the S protein of HCoV-OC43 interacts with N protein and that cell penetrating Spike CD peptide inhibits virus protein expression and replication of HCoV-OC43. The interaction between HCoV-OC43 S and N proteins were recapitulated with a recombinant HCoV-OC43 Spike CD fusion protein and a recombinant HCoV-OC43 N fusion protein *in vitro*. By producing an anti-HCoV-OC43 N protein-specific monoclonal antibody, we established a virus detection system based on the interaction between recombinant Spike CD and N protein of HCoV-OC43. We suggest that the interaction between Spike CD and N protein is conserved in coronaviruses and therefore could be a target for therapeutics against both novel coronavirus and its variants.

## Introduction

Coronaviruses (CoVs) cause infections of the upper respiratory tract that range in severity from mild to lethal. CoVs did not attract much scientific attention until the outbreak of severe acute respiratory syndrome (SARS) in 2002 (1). CoVs are enveloped, non-segmented positive-sense RNA viruses belonging to the *Coronaviridae* family. CoVs are divided into four genera including *Alphacoronaviruses*, *Betacoronaviruses*, *Gammacoronaviruses*, and *Deltacoronaviruses* by International Committee for Taxonomy of Viruses (2, 3). CoVs infect many different hosts, including bats, cattle, avians, swine, and humans. Currently, there are seven reported human coronaviruses (HCoVs). Two HCoVs, HCoV-229E and HCoV-NL63, are classified in the *Alphacoronavirus* genera, whereas the other five HCoVs are in the *Betacoronaviruses* genera. Within the *Betacoronaviruses* genera, HCoVs are divided by lineage, as HCoV-HKU1 and HCoV-OC43 belong to lineage A, severe acute respiratory syndrome coronavirus (SARS-CoV) and SARS-CoV-2 belong to lineage B, and Middle East respiratory syndrome coronavirus (MERS-CoV) belongs to lineage C (4, 5). Highly pathogenic coronaviruses belonging to *Betacoronaviruses* emerged in 2002 (SARS-CoV), in 2012 (MERS-CoV), and in 2019 (SARS-CoV-2). SARS-CoV-2 is now the causative agent of the ongoing COVID-19 pandemic, highlighting the importance of studying HCoVs (6).

All HCoVs contain four major structural protein complexes: spike glycoprotein (S protein), nucleocapsid phosphoprotein (N protein), Membrane glycoprotein (M protein) and small envelope glycoprotein (E protein) (7). Many studies have explored the mechanism of receptor-mediated host cell entry by S protein binding of *Betacoronaviruses*. SARS-CoV and SARS-CoV-2 S protein binds to angiotensin-converting enzyme 2 (ACE2) (8, 9), MERS-CoV S protein binds to dipeptidyl peptidase 4 (DPP4) (10), and HCoV-OC43 S protein binds to 9-O-acetylated sialic acid (11). Taken together, these results suggest that S proteins are ideal targets for therapeutic agents against pathogenic coronaviruses.

In addition to S protein, N protein is considered another potential therapeutic target against coronavirus infection (12). N protein dimers bind to viral RNA and are essential for CoV replication (13). N protein consists of two major domains: the N-terminal domain (NTD) and C-terminal domain (CTD). Many investigators have characterized the viral RNA binding of N proteins in SARS-CoV (14, 15), SARS-CoV-2 (16, 17), MERS-CoV (18, 19), and HCoV-OC43 (20) through structural analysis. Interactions between M and N proteins play an important role in the assembly of SARS-CoV (21), SARS-CoV-2 (22), MERS-CoV (23), and HCoV-OC43 (24). Recently, we suggested that a direct interaction between the S protein and N protein contributes to the replication of MERS-CoV and SARS-CoV-2 (25). However, interactions between S and N proteins have yet to be identified in other coronaviruses.

Here, we identified a direct interaction between the S and N proteins in HCoV-OC43-infected cells. We found that inhibition of this interaction by synthetic cell-penetrating peptide corresponding to the C-terminal domain of S protein (Spike CD) of HCoV-OC43 reduced the production of HCoV-OC43 viruses in infected cells. Thus, therapeutic agents targeting the interaction of S and N proteins could be effective against HCoV-OC43.

## Materials and Methods

### Cell Culture

Vero cells originating from African green monkey kidneys were obtained from the Korean Cell Line Bank (Seoul, Korea). The cells were cultured in Dulbecco’s modified Eagle’s medium (DMEM, ATCC, Manassas, VA, USA) containing 10% fetal bovine serum (FBS, Thermo Fisher Scientific, Waltham, MA, USA), penicillin (100 U/ml), and streptomycin (100 µg/ml). Cells were maintained in 95% atmospheric air and 5% CO_2_ at 37°C.

### Virus Amplification

HCoV-OC43 (KBPV-VR-8) was provided by the Korea Bank for Pathogenic Viruses (College of Medicine, Korea University, Seoul, Korea). The virus amplification was carried out as described previously [26]. Briefly, Vero cells cultured in six-well plates (3 × 10^5^ cells/well) were infected with HCoV-OC43 suspended in phosphate-buffered saline (PBS) at a multiplicity of infection (MOI) of 0.03. After incubation for 1 h at 37°C with shaking every 10 min, the cells were cultured in DMEM containing 2% FBS at 37°C in a 5% CO_2_ incubator. At 4 days post-infection, the cell culture supernatants were harvested and centrifuged for 10 min at 3,000 rpm. Supernatants were collected and stored at -70°C. HCoV-OC43 amplification was performed under biosafety level 2 (BSL-2) conditions in the Translational Research Center of Hallym University. Both MERS-CoV (MERS-CoV/KOR/KNIH/002_05_2015) and SARS-CoV-2 S clade (hCoV-19/South Korea/KCDC03/2020, NCCP43326) were provided by the National Culture Collection for Pathogens (Osong, Korea). MERS-CoV and SARS-CoV-2 amplification and cell culture procedures were performed according to biosafety level 3 (BSL-3) conditions in the Hallym Clinical and Translational Science Institute in accordance with the recommendations of the Institutional Biosafety Committee of Hallym University (Permit no. Hallym2020-12).

### Plaque Assay

HCoV-OC43 titers were quantified by plaque assay as described previously (26, 27). Briefly, Vero cells (7 × 10^5^ cells/well) were cultured in 6-well plates (Corning, NY, USA) for 18 h. The cells were washed with PBS and inoculated with tenfold serial dilutions of the virus-infected culture supernatants in PBS at 37°C. After 1 h of infection, the medium was aspirated from the wells and 3 ml DMEM/F12 (Thermo Fisher Scientific) mixed with 2% Oxoid agar was added to the wells. At 5 days post-infection, the overlay medium was removed, and plaque formation was visualized by staining with 0.1% crystal violet.

### Peptides Synthesis

To analyze the interaction between the S and N proteins, Spike protein C-terminal domain (Spike CD HCoV-OC43) peptide was synthesized based on the HCoV-OC43 S protein sequence (YP_009555241.1: Spike CD HCoV-OC43, ^1320^CCTGCGTSCFKKCGGCCDDYTGYQELVIKTSHDD^1353^. The peptide was conjugated with nine D-arginine residues at the N-terminus (R-Spike CD HCoV-OC43) to penetrate host cells. A negative control cell-penetrating peptide (R-CP-1) was synthesized by Anygen Co., Ltd. (Gwang Ju, South Korea) to contain nine D-arginine residues (NH_2_-d-RRRRRRRRR-AQARRKNYGQLDIFP-COOH) (28).

### Antibodies

Antibodies against HCoV-OC43 N (Mouse anti-HCoV-OC43 N monoclonal Ab, Catalog No. LS-C79764) and S protein (Rabbit anti-HCoV-OC43 S Ab, Catalog No. LS-C371066) were purchased from LifeSpan BioSciences (Seattle, WA, USA). Both the peroxidase-conjugated streptavidin (Catalog No. S5512) and antibody for β-actin were obtained from Sigma-Aldrich (St. Louis, MO, USA). The antibody against the Fc domain of human IgG (Catalog No. 790-035-098) was obtained from Jackson ImmunoResearch Laboratories (West Grove, PA, USA).

### HCoV-OC43 Infection and Western Blotting

Vero cells were grown in six-well plates at a density of 3 × 10^5^ cells/well. The cells were cultured for 18 h and infected with HCoV-OC43 (MOI of 0.1) in PBS for 1 h in a 5% CO_2_ incubator at 37°C. After infection, the supernatants were removed, and the culture medium was changed with DMEM containing 2% FBS. After 3 days of infection, HCoV-OC43-infected Vero cells were lysed in cell lysis buffer (10 mM HEPES, 150 mM NaCl, 5 mM EDTA, 100 mM NaF, 2 mM Na_3_VO_4_, protease inhibitor cocktail, and 1% NP-40) for 30 min at 4°C. Cell lysates were collected by centrifugation at 14,000 rpm at 4°C for 20 min. Equal amounts of proteins from each cell lysate were resolved on 4–12% Bis-Tris gradient gels (Bolt™ 4–12% Bis-Tris Plus gel, Thermo Fisher Scientific) and transferred onto nitrocellulose membranes. The membranes were blocked with 3% BSA, incubated with either anti-HCoV-OC43 N monoclonal Ab or anti-HCoV-OC43 S Ab overnight at 4°C, then subsequently incubated with horseradish peroxidase (HRP)-conjugated secondary antibodies. Protein bands were developed using enhanced chemiluminescence reagent (Thermo Fisher Scientific) and analyzed with ChemiDoc (Bio-Rad, Hercules, CA, USA).

### Co-Immunoprecipitation

The HCoV-OC43-infected cell lysates were incubated with anti-HCoV-OC43 S Ab for 3 h at 4°C. Protein A beads (CaptivA™ PriMAB 52% (v/v) slurry, Repligen, Waltham, MA, USA) were added into the cell lysates. After 1 h incubation at 4°C, the immunocomplexes were collected by centrifugation, then separated by 4–12% gradient SDS-PAGE (Thermo Fisher Scientific) and subjected to western blotting analysis using either anti-HCoV-OC43 N monoclonal Ab or anti-HCoV-OC43 S Ab.

### Confocal Images

To analyze the inhibitory effect of the HCoV-OC43 Spike CD peptide on HCoV-OC43 S protein and N protein expression, confocal images were analyzed as described previously (29). Vero cells (5 × 10^4^ cells/well) were cultivated on coverslips on 12-well plates for 18 h. The cells were infected with HCoV-OC43 (MOI of 0.1) in PBS for 1 h in a 5% CO_2_ incubator at 37°C. After infection, the culture medium was changed to DMEM containing 2% FBS. The cells were then treated 6 h post-infection with R-Spike CD HCoV-OC43 peptide or Spike CD HCoV-OC43 peptide. After 48 h incubation in a 5% CO_2_ incubator at 37°C, the cells were fixed with 4% paraformaldehyde then permeabilized with Tween-20 in PBS (PBST) containing 1% bovine serum albumin (BSA). The permeabilized cells were incubated with anti-HCoV-OC43 N monoclonal Ab (1:5,000 dilution) or anti-HCoV-OC43 S Ab (1:200 dilution) for 2 h, washed with PBST containing 1% BSA, and incubated for 1 h with Alexa Fluor 488-conjugated secondary antibody (Thermo Fisher Scientific). Hoechst 33258 was used for nuclei staining. The cells were observed using a Carl Zeiss LSM710 microscope.

### Inhibition of the HCoV-OC43 Spike CD Peptide on HCoV-OC43 Replication

To investigate the effect of HCoV-OC43 Spike CD peptide on HCoV-OC43 replication, we also performed plaque reduction assay as described previously (29, 30). Vero cells (7 × 10^5^ cells/well) were cultured on six-well plates for 18 h. HCoV-OC43 (200 pfu) was mixed with two-fold serially diluted R-Spike CD HCoV-OC43 peptide, Spike CD HCoV-OC43 peptide or R-CP-1 peptide in PBS. Vero cells on six-well plates were washed with PBS and then the diluted R-Spike CD HCoV-OC43 peptide, Spike CD HCoV-OC43 peptide, or R-CP-1 peptide mixtures was added to the Vero cells. The cells were incubated for 1 h in a 5% CO_2_ incubator at 37°C, shaking at 15–20-min intervals. After incubation, the supernatants were removed, and the virus replication was quantified using the previously described plaque assay.

### Cell Viability Assays

To examine the cytotoxic effect of the cell-penetrating peptides in cells, Vero cells (5 × 10^3^ cells/well) were cultured on 96-well plates in DMEM containing 10% FBS. After cultivation for 18 h, the culture media was changed to DMEM containing 2% FBS and cultured for 6 h. The cells were then incubated with two-fold serially diluted R-Spike CD HCoV-OC43 peptide, Spike CD HCoV-OC43 peptide or R-CP-1 peptide for 48 h. Lastly, the cells were incubated with Cell Counting Kit-8 (CCK-8) solution (Dojindo Molecular Technologies, Rockville, MD, USA) for 2 h at 37°C. Soluble formazan was measured by absorbance at 450 nm using a microplate reader (Thermo Fisher Scientific, Ratastie, Finland).

### Construction and Expression of Biotin Peptide-6xHis-Tagged HCoV-OC43 N Protein

A fusion gene encoding a biotin peptide-6xHis-tagged (NSGSLHHILDAQKMVWNHR-DRNLPPLAPLGPHHHHHH) HCoV-OC43 N protein (GenBank ID: NC006213.1, nucleotide number 29079-30425, protein YP_009555245.1) was synthesized (Bioneer, Daejeon, Korea) with restriction enzyme sites (Not I and Kpn I) included at 5′ and 3′ ends, respectively. The biotin peptide sequence was recognized by the *Escherichia coli* biotin holoenzyme synthetase, BirA (31–33). The synthesized fusion gene was inserted into the modified pcDNA 3.4 expression vector (Invitrogen, Waltham, MA, USA) containing IL-2 signal sequences (pcDNA3.4-HCoV-OC43 N protein-Biotin-His_6_) for mammalian cell expression. The HCoV-OC43 N protein-Biotin-His_6_ was expressed in ExpiCHO cells (catalog No. A29133, Thermo Fisher Scientific) with modified expression vector containing BirA (pTT3-secreted BirA-8xHis, Catalog No. #32408; Addgene, Watertown, MA, USA). The HCoV-OC43 N protein-Biotin-His_6_ was purified from ExpiCHO culture supernatants after 14 days of cell culture at 32°C using Ni-NTA-agarose (Qiagen) chromatography. The expression and purification of HCoV-OC43 N protein-Biotin-His_6_ was confirmed by SDS-PAGE and western blot analysis with the anti-His-tag antibody.

### Construction and Expression of HCoV-OC43 Spike CD-Human Fc Fusion Proteins

Fusion gene encoding HCoV-OC43 Spike CD (GenBank ID: NC_006213, nucleotide number 27600-27701, protein YP_009555241.1) and human IgG1 Fc domain (GenBank ID: AK123800.1. protein) was synthesized (Bioneer, Daejeon, Korea), including restriction enzyme sites (Not I and Kpn I) at 5′ and 3′ ends, respectively. This fusion gene was inserted into the modified pcDNA 3.4 expression vector containing IL-2 signal sequences (pcDNA3.4-HCoV-OC43 Spike CD-Fc) for mammalian cell expression. The HCoV-OC43 Spike CD-Fc fusion protein was expressed using the in Gibco™ ExpiCHO™ Expression System Kit according to the manufacturer’s instructions. At 14 days after ExpiCHO culturing at 32°C, the HCoV-OC43 Spike CD-Fc fusion protein was purified using Protein A affinity chromatography. The purity of the HCoV-OC43 Spike CD-Fc fusion protein was evaluated using SDS-PAGE analysis.

### Mice Immunization

Monoclonal antibodies against HCoV-OC43 N protein were produced using 4-week-old BALB/c mice (female, H-2^b^) provided by Nara-Biotec (Seoul, Korea). A mixture of recombinant HCoV-OC43 N-Bio-His_6_ protein (50 μg) and MB-ODN 4531(O) (CpG-DNA, (50 μg) was encapsulated in the DOPE : CHEMS complex (molar ratio of 1:1) as reported previously (34). BALB/c mice were intraperitoneally immunized with the HCoV-OC43 N protein complex three times at 14-day intervals. Animal care and experimental protocols were approved by the Hallym University Institutional Animal Care and Use Committee (Hallym2021-12).

### Production of Mouse Monoclonal Antibody Against HCoV-OC43 N Protein

Mouse SP2/0 myeloma cells were fused with splenocytes derived from HCoV-OC43 N protein-immunized mice using polyethylene glycol solution (PEG, Sigma-Aldrich) to generate hybridoma cells. Hybridoma cells were obtained and cloned in HAT medium (Sigma-Aldrich) and HT medium (Sigma-Aldrich) according to the standard hybridoma production method (34). Hybridoma cells producing mouse monoclonal antibody against HCoV-OC43 N protein were injected into the peritoneal cavity of BALB/c mice. Then, ascites was collected from the peritoneal cavity of BALB/c mice, and monoclonal antibody against HCoV-OC43 N protein was purified using Protein A column chromatography.

### Antigen-Specific Ig ELISA

Streptavidin (2 μg/well) in ELISA coating buffer (0.1 M carbonate buffer, pH 9.6) was coated in 96-we1l immunoplates (Thermo Fisher Scientific) overnight at 4°C. The plates were blocked with PBS supplemented with PBST containing 3% BSA. Recombinant HCoV-OC43 N-Bio-His_6_ protein (2 μg/well) was added to each well for 2 h and then mouse sera, hybridoma culture supernatants, ascites, or purified monoclonal antibody solution were added to each well to measure HCoV-OC43 N protein-specific antibody levels by standard ELISA as described previously (34). The subclass of the monoclonal antibody was identified with HRP-conjugated anti-mouse IgG (each subclass) antibodies (Southern Biotech, Birmingham, AL, USA).

### Measurement of Monoclonal Antibody Binding Affinity by ELISA

The binding affinity of the purified HCoV-OC43 N protein-specific monoclonal antibody was measured by ELISA as described previously (35). Briefly, streptavidin (2 μg/well) was coated in 96-well immunoplates (Thermo Fisher Scientific), and then recombinant HCoV-OC43 N-Bio-His_6_ protein (3 μg/well) was added into each well along with serially diluted (1:5) monoclonal antibody (clone 1C7D7 mAb) in PBST. The monoclonal antibody binding to HCoV-OC43 N protein was detected with HRP-conjugated anti-mouse IgG antibody (1:5,000 dilution, Catalog No. 715-035-150, Jackson ImmunoResearch Laboratories) and tetramethylbenzidine (TMB) peroxidase substrate (Kirkegaard and Perry Laboratories, Gaithersburg, MD, USA). The absorbance of each well was measured at 450 nm using the Spectra Max 250 microplate reader (Molecular Devices, San Jose, CA, USA). SigmaPlot was used to determine the EC_50_ value (27). To evaluate the specificity of the monoclonal antibody, recombinant MERS-CoV N protein-Bio-His_6_ and SARS-CoV-2 N protein-Bio-His_6_ (35) were analyzed as a control.

### Preparation of Virus-Infected Cell Lysates

To investigate the specificity of monoclonal antibody (clone 1C7D7 mAb) obtained against HCoV-OC43 N protein, cell lysates were prepared from Vero cells infected with SARS-CoV-2, MERS-CoV, or HCoV-OC43 as described previously (26). Vero cells (3 × 10^5^ cells) were cultured in six-well plates for 18 h. After washing the cells with PBS, each virus (0.1 MOI) was inoculated into each well in PBS and then incubated for 1 h in a 5% CO_2_ incubator at 37°C with shaking every 15 min. Cells were washed with PBS then cultured for 72 h at 37°C in a CO_2_ incubator in either 2 ml of DMEM/F12 for MERS-CoV or DMEM medium containing 2% FBS for SARS-CoV-2 and HCoV-OC43. After culturing, the cells were washed with PBS and lysed for 30 min with cell lysis buffer (10 mM HEPES, 150 mM NaCl, 5 mM EDTA, 100 mM NaF, 2 mM Na_3_VO_4_, protease inhibitor cocktail, 1% NP-40). The cell lysates were centrifuged at 13,000 rpm at 4°C for 10 min, and supernatants were collected and stored at -80°C.

### Detection of HCoV-OC43 in Cell Culture Supernatants by ELISA

The interaction of HCoV-OC43 Spike CD and HCoV-OC43 N protein was measured by ELISA as described previously (35). Briefly, HCoV-OC43 N protein-specific monoclonal antibody (clone 1C7D7 mAb, 3 μg/well) was coated in 96-well immunoplates overnight at 4°C, then blocked with PBST containing 1% BSA. HCoV-OC43 viral particles in cell culture supernatants were lysed with cell lysis buffer, serially diluted (1:3) in PBST, and added to the wells of the plate. After incubation for 2 h at room temperature, recombinant Fc domain or HCoV-OC43 Spike CD-Fc fusion protein were added along with goat anti-human IgG Fc antibody conjugated with HRP (1:5000 dilution, Catalog No.109-035-008, Jackson ImmunoResearch Laboratories). After developing with TMB peroxidase substrate, the amount of HCoV-OC43 N protein in each well was quantified using a Spectra Max 250 microplate reader (absorbance at 450 nm).

### Evaluation of the Interaction Between HCoV-OC43 Spike CD-Human Fc Fusion Protein and HCoV-OC43 N Protein

To analyze the interaction of HCoV-OC43 Spike CD and HCoV-OC43 N protein, we designed a “bait and prey” ELISA, described previously (35). Briefly, streptavidin (2 μg/well) was coated in 96-well immunoplates then blocked with PBST containing 1% BSA. HCoV-OC43 N protein-Biotin-His_6_ (3 μg/well) was added to each well and incubated for 2 h at room temperature. Recombinant MERS-CoV N protein-Bio-His_6_ and SARS-CoV-2 N protein-Bio-His_6_ (35) were used as a bait control to confirm the specificity of the interaction. After washing with PBST, HCoV-OC43 Spike CD-Fc fusion protein serially diluted to 1:3 was added to each well in PBST, then incubated for 2 h at room temperature. After washing with PBST, goat anti-human IgG Fc antibody conjugated with HRP (1:5000 dilution) was added to each well. Interactions between HCoV-OC43 Spike CD-human Fc fusion protein and HCoV-OC43 N protein were measured by developing with TMB peroxidase substrate. To analyze the interaction of HCoV-OC43 Spike CD-human Fc fusion protein and HCoV-OC43 N protein in the viral particles, cell culture supernatants containing HCoV-OC43 were lysed with cell lysis buffer and then added to the recombinant Fc domain or HCoV-OC43 Spike CD-Fc protein. After incubation for 2 h at 4°C, Protein A beads were added, and the immunocomplexes were collected and then subjected to western blotting analysis using anti-HCoV-OC43 N monoclonal Ab (clone 1C7D7 mAb).

### Statistical Analysis

Results are shown as the mean ± standard deviation. Differences between the samples were analyzed using an unpaired, two-tailed nonparametric t-test of significance (Instat; GraphPad Inc., San Diego, CA, USA). p-values < 0.05 were considered statistically significant.

## Results

### S Protein and N Protein Interact With One Another in HCoV-OC43

**Figure 1:**
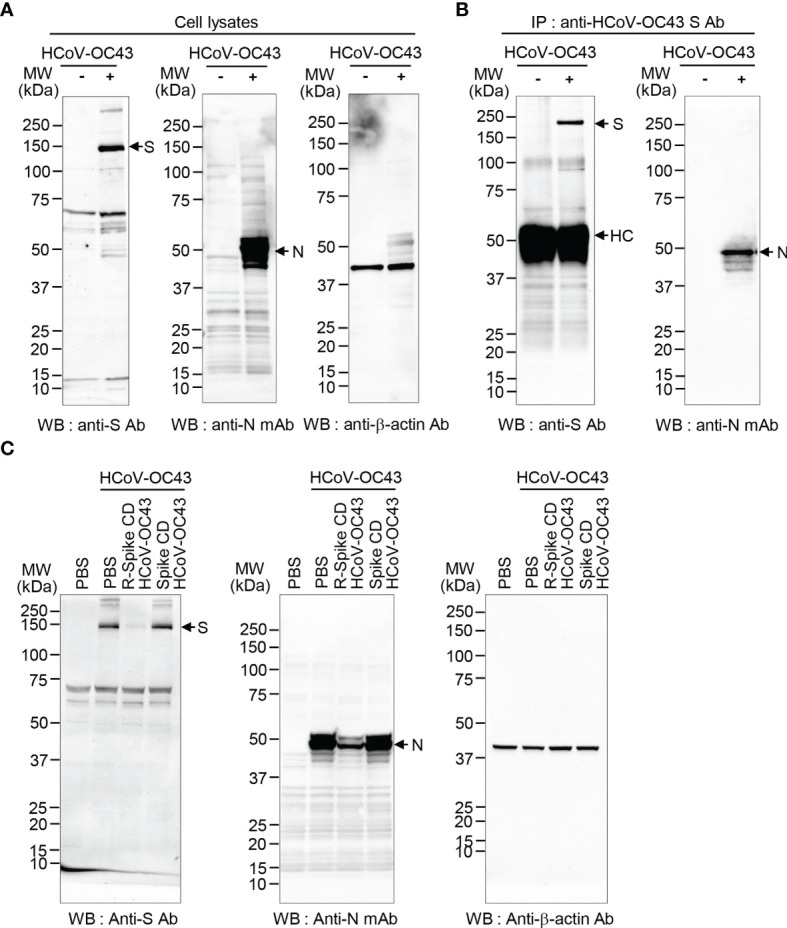
HCoV-OC43 S protein and N protein interact. **(A)** Expression of S and N proteins in HCoV-OC43-infected cells. Lysates of uninfected and HCoV-OC43 (0.1 MOI)-infected Vero cells were prepared and analyzed by western blotting with rabbit anti-HCoV-OC43 S antibody or mouse anti-HCoV-OC43 N monoclonal antibody. Anti-β-actin antibody was used as a loading control. S, spike protein. N, nucleocapsid protein. **(B)** Co-immunoprecipitation of N and S proteins. Equal amounts of the lysates (150 μg protein) were immunoprecipitated with anti-HCoV-OC43 S antibody. The immunocomplexes were analyzed by western blotting with rabbit anti-HCoV-OC43 S antibody or mouse anti-HCoV-OC43 N monoclonal antibody. HC, heavy chain. Ab, antibody. **(C)** HCoV-OC43 S and N protein expression is reduced by R-Spike CD HCoV-OC43 peptide. Vero cells were infected with HCoV-OC43 (0.1 MOI). After 6 h, the cells were treated with 2 μM of R-Spike CD HCoV-OC43 peptide or Spike CD HCoV-OC43 peptide. Cell lysates were prepared at 48 h after infection and analyzed by western blotting with the indicated antibodies.

In our previous work, we reported that Spike CD and N proteins directly interact during virus assembly of MERS-CoV and SARS-CoV-2 (25). Therefore, we hypothesized that the interaction between S protein and N protein might be conserved across coronaviruses. Here, we identified an interaction between S and N proteins of HCoV-OC43 through the immunocomplex analysis after immunoprecipitation using lysates of HCoV-OC43-infected Vero cells and a polyclonal antibody against the HCoV-OC43 S protein (anti-HCoV-OC43 S Ab). HCoV-OC43 S and N proteins were expressed in infected cells and N protein was coimmunoprecipitated with S protein (**Figures 1A, B**).

### Cell-Penetrating Spike CD HCoV-OC43 Peptide Reduces HCoV-OC43 S and N Protein Production

**Figure 2:**
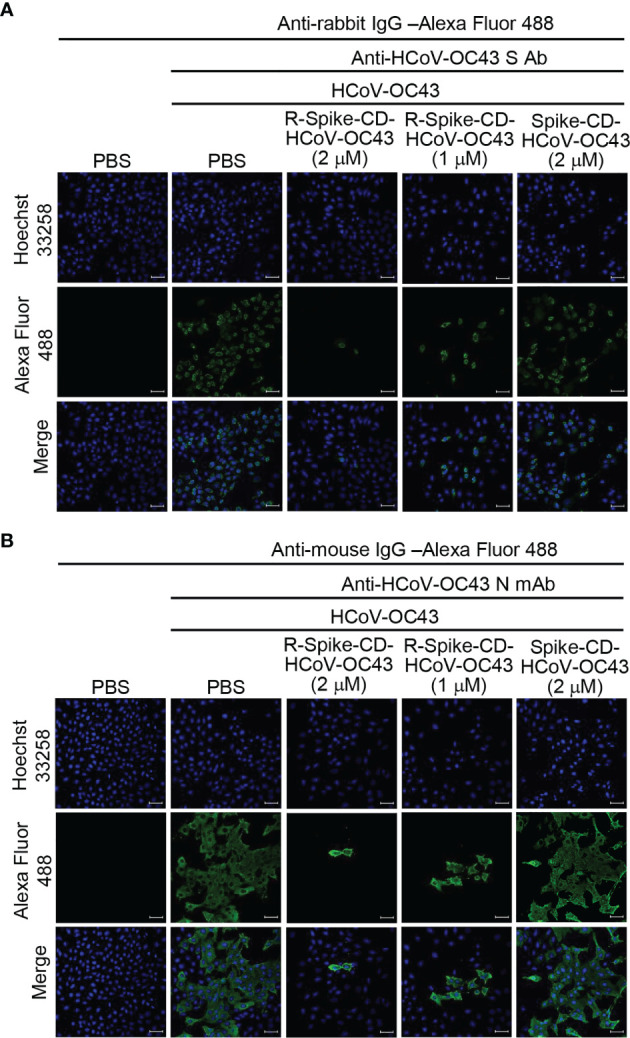
R-Spike CD HCoV-OC43 peptide reduces N and S protein production. Vero cells were infected with HCoV-OC43 (0.1 MOI). After 6 h, the cells were treated with PBS control or R-Spike CD HCoV-OC43 peptide (1 or 2 μM) or Spike CD HCoV-OC43 peptide (2 μM). The cells were cultured for 48 h and then stained with rabbit anti-HCoV-OC43 S antibody **(A)** or mouse anti-HCoV-OC43 N monoclonal antibody and then **(B)** Alexa Fluor 488-conjugated secondary antibodies. The expression of HCoV-OC43 S protein and N protein were analyzed by confocal microscopy. Expression of S and N proteins measured by the fluorescence was decreased in the presence of R-Spike CD HCoV-OC43 peptide. Scale bar, 20 μm.

Previously, we found that interaction between S and N proteins of MERS-CoV and SARS-CoV-2 was reduced by each cell-penetrating Spike CD peptide because the cell-penetrating Spike CD peptides interfered with the intracellular production of MERS-CoV and SARS-CoV-2 S and N proteins (25). Therefore, we investigated the effect of the cell-penetrating Spike CD HCoV-OC43 peptide (R-Spike CD HCoV-OC43) on the production of HCoV-OC43 S and N proteins. We found that S and N protein levels in HCoV-OC43-infected cells were consistently reduced by R-Spike CD HCoV-OC43 peptide compared to PBS control or cell-impermeable Spike CD HCoV-OC43 peptide (**Figure 1C**). We confirmed the inhibitory effect of R-Spike CD HCoV-OC43 peptide on S and N protein production by confocal image analysis. Confocal images showed remarkably reduced S and N protein production by R-Spike CD peptide in a concentration-dependent manner compared to PBS or Spike CD peptide controls (**Figure 2**).

### Cell-Penetrating HCoV-OC43 Spike CD Peptide Inhibits HCoV-OC43 Replication

**Figure 3:**
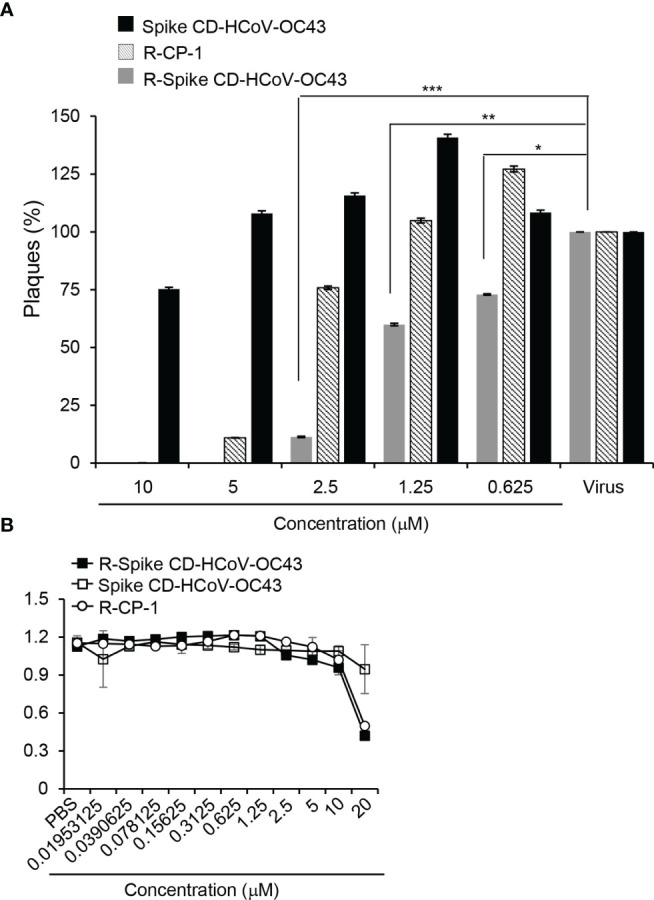
R-Spike CD HCoV-OC43 peptide reduces HCoV-OC43 replication without cytotoxicity. **(A)** Vero cells in 6-well plates were infected with HCoV-OC43 (200 pfu/well) with indicated concentration of R-Spike CD HCoV-OC43 peptide, Spike CD HCoV-OC43 peptide, or R-CP-1 peptide (n=3). Plaques formed for 5 days were counted and compared between the peptide-treated samples and control samples treated with virus only. The plaque formation was reduced by R-Spike CD HCoV-OC43 peptide. **(B)** Vero cells were cultured with the indicated concentrations of cell-penetrating peptides for 48 h and subjected to viability assay. There was no cytotoxic effect of the peptides at concentrations below 10 μM. *p < 0.05, **p < 0.01, ***p < 0.001.

To investigate the effect of cell penetration by Spike CD HCoV-OC43 peptide on the replication of HCoV-OC43, Vero cells were infected with HCoV-OC43 in combination with R-Spike CD HCoV-OC43, Spike CD HCoV-OC43 or R-CP-1. Plaque formation assays revealed that HCoV-OC43 replication was significantly reduced by the treatment of R-Spike HCoV-OC43 peptide compared to Spike HCoV-OC43 peptide treatment (**Figure 3A**). Treatment with control R-CP-1 had some inhibitory effect at 2.5 μM and significant inhibitory effects at 5 or 10 μM on plaque formation, suggesting nonspecific effects of the cell-permeable peptides especially at high concentrations. To investigate potential side effects of cell-penetrating Spike CD peptides, we measured the cytotoxicity of R-Spike CD HCoV-OC43, Spike CD HCoV-OC43 or R-CP-1 in Vero cells. Cytotoxic effects of the cell-penetrating peptides were not observed at concentrations below 10 μM in Vero cells (**Figure 3B**), suggesting that there are no side effects of cell-penetrating Spike CD peptides at the concentration we used for functional assays in **Figures 1**, **2** (2 μM). Our results showed that inhibition of the interaction between the Spike CD and the N protein using a cell-permeable Spike CD HCoV-OC43 peptide reduces HCoV-OC43 replication.

### Recombinant HCoV-OC43 Spike CD Fusion Protein Interacts With Recombinant HCoV-OC43 N Fusion Protein

**Figure 4:**
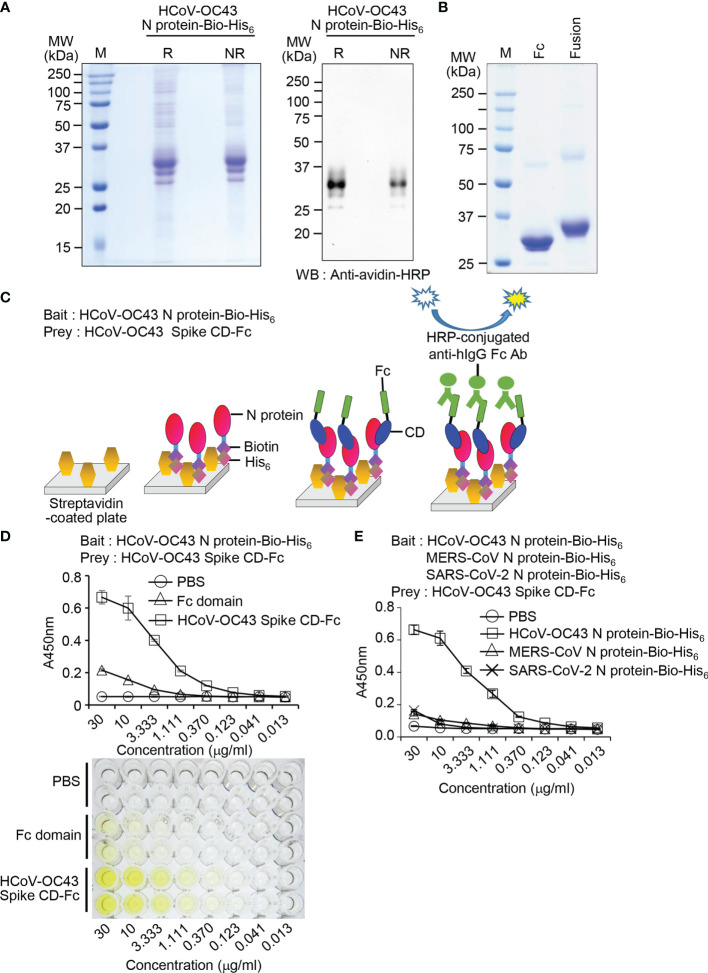
The Spike CD-Fc fusion protein interacts with N protein-Bio-His_6_ recombinant protein in HCoV-OC43-infected cells. **(A)** Expression of biotin peptide-6×His-tagged HCoV-OC43 N protein (HCoV-OC43 N protein-Bio-His_6_). The purified recombinant protein was analyzed by SDS-PAGE (left) and western blotting with peroxidase-conjugated streptavidin (right). R, reducing condition; NR, non-reducing condition. **(B)** Expression of HCoV-OC43 Spike CD-Fc. The purified recombinant Fc domain protein and the HCoV-OC43 Spike CD-Fc fusion protein were analyzed by SDS-PAGE and Coomassie blue staining. **(C)** Schematic of the bait and prey assay system. **(D, E)** The amount of HCoV-OC43 Spike CD-Fc bound to HCoV-OC43 N protein-Bio-His_6_ recombinant protein in the wells was determined by ELISA. PBS, Fc domain, MERS-CoV N protein-Bio-His_6_ and SARS-CoV-2 N protein-Bio-His_6_ were used as a bait control. HCoV-OC43 Spike CD-Fc specifically interacted with HCoV-OC43 N protein-Bio-His_6_.

Previously, we developed an ELISA-based “bait and prey” system to confirm direct interaction between SARS-CoV-2 Spike CD and N protein using recombinant fusion proteins (35). Here, we used this ELISA-based “bait and prey” system to confirm the interaction between HCoV-OC43 Spike CD and N protein *in vitro*. To do this, we expressed and purified recombinant HCoV-OC43 N protein-Bio-His_6_ fusion protein (**Figure 4A**) and HCoV-OC43 Spike CD-Fc fusion protein (**Figure 4B**). Expected molecular mass of the recombinant Fc proteins are 25.5 kDa and 29.5 kDa, respectively, and most of the purified recombinant proteins showed major single bands with similar mass. However, there were minor protein bands with about two-fold higher molecular mass suggesting partial formation of dimers (**Figure 4B**). We designed a bait and prey assay system employing streptavidin, HCoV-OC43 N protein-Bio-His_6_, HCoV-OC43 Spike CD-Fc, and anti-human IgG Fc antibody conjugated with HRP (**Figure 4C**). The ELISA results showed that HCoV-OC43 Spike CD-Fc binds to HCoV-OC43 N protein-Bio-His_6_ in a concentration-dependent manner. There was weak binding of N protein-Bio-His_6_ to the Fc control at high concentrations and no binding in PBS control (**Figure 4D**). To further confirm the specificity of the interaction, we performed the same experiments with MERS-CoV N protein-Bio-His_6_ and SARS-CoV-2 N protein-Bio-His_6_ as a bait and found only weak binding of HCoV-OC43 Spike CD-Fc to other virus recombinant N proteins (**Figure 4E**). Taken together, our results support the idea that the Spike CD and the N protein of HCoV-OC43 interact specifically and directly with one another and suggest that our bait and prey system can be used to quantitatively assess this interaction *in vitro*.

### Production of HCoV-OC43 N Protein-Specific Monoclonal Antibody and Detection of the Interaction Between Recombinant HCoV-OC43 Spike CD Fusion Protein and N Protein of HCoV-OC43 Particles

**Figure 5:**
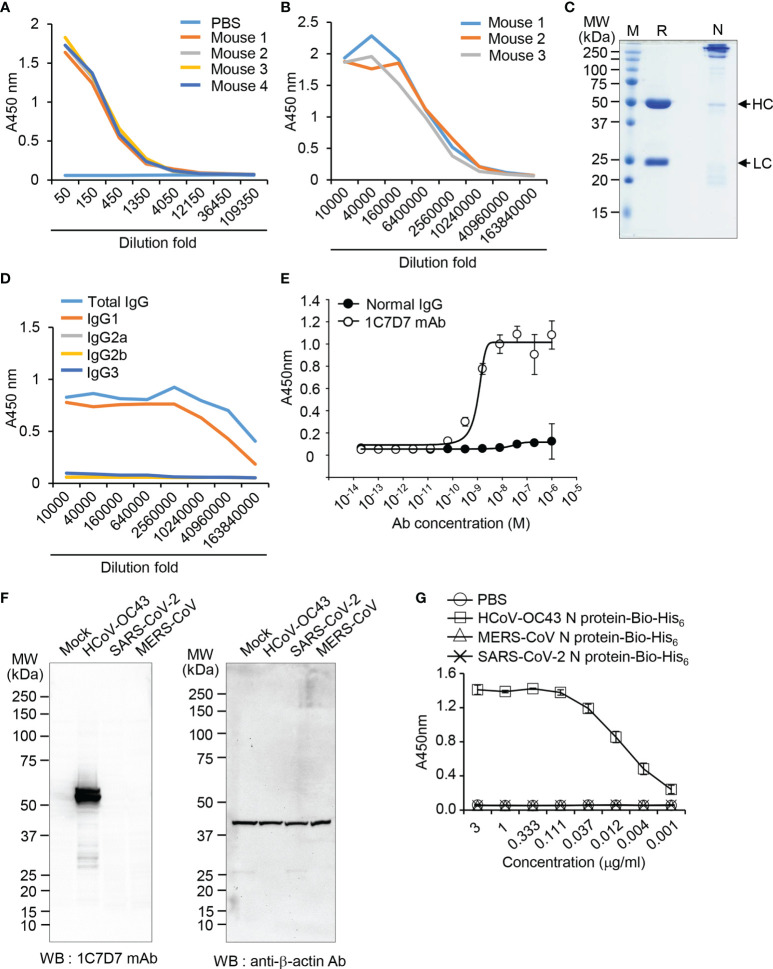
Production and characterization of the anti-HCoV-OC43 N protein-specific monoclonal antibody. **(A)** ELISA was performed to determine the production of recombinant HCoV-OC43 N-Bio-His_6_ protein-specific antibody in the immunized mice. **(B)** Ascites was collected from mice injected with cloned hybridoma cells (1C7D7). Production of recombinant HCoV-OC43 N-Bio-His_6_ protein-specific antibody in the ascites was determined by ELISA. **(C)** The purified monoclonal antibody in the ascitic fluid was analyzed by SDS-PAGE. HC, heavy chain. LC, light chain. **(D)** Subclasses of the purified monoclonal antibody were determined by ELISA. **(E)** Binding of the purified monoclonal antibody (clone 1C7D7 mAb) to recombinant HCoV-OC43 N-Bio-His_6_ protein. **(F)** Mock-infected or HCoV-OC43-, MERS-CoV- or SARS-CoV-2-infected cell lysates were analyzed by western blotting with the purified monoclonal antibody. Anti-β-actin antibody was used as a control. The purified monoclonal antibody specifically recognizes N protein of HCoV-OC43. **(G)** Recombinant HCoV-OC43 N-Bio-His_6_, SARS-CoV-2 N-Bio-His_6_ or MERS-CoV N-Bio-His_6_ proteins were captured on streptavidin-coated 96-well immunoplates and then incubated with the purified monoclonal antibody for ELISA. Th antibody was specific to recombinant HCoV-OC43 N-Bio-His_6_.

**Figure 6:**
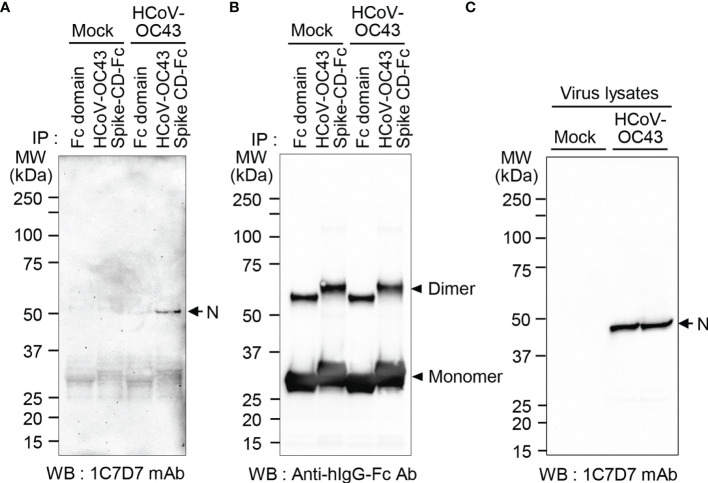
N protein in HCoV-OC43 particles interacts with HCoV-OC43 Spike CD. Cell culture supernatants containing HCoV-OC43 were lysed with cell lysis buffer and incubated with Fc or HCoV-OC43 Spike CD-Fc. Fc-bound proteins were pulled down with Protein A beads and subjected to western blotting using anti-HCoV-OC43 N monoclonal antibody (clone 1C7D7 mAb) **(A)** or anti-hIgG Fc antibody **(B)**. Virus lysates were analyzed using anti-HCoV-OC43 N monoclonal antibody (clone 1C7D7 mAb) as a loading control **(C)**.

To detect the N protein of HCoV-OC43 particles, we produced a monoclonal antibody against N protein of HCoV-OC43 using recombinant HCoV-OC43 N protein-Bio-His_6_ and CpG-DNA co-encapsulated in a liposome (DOPE : CHEMS) as described previously (34). First, we confirmed production of antibody against recombinant HCoV-OC43 N protein from immunized mice (**Figure 5A**). Second, one hybridoma clone (1C7D7) producing HCoV-OC43 N protein-specific monoclonal antibody was selected from several clones and the HCoV-OC43 N protein-specific monoclonal antibody (1C7D7 mAb) was purified from the ascitic fluids of mice injected with hybridoma cells (**Figures 5B, C**). Third, we characterized the purified monoclonal antibody. The IgG subclass of the purified monoclonal antibody was IgG1 (**Figure 5D**). Binding of the monoclonal antibody to recombinant HCoV-OC43 N protein-Bio-His_6_ was measured by ELISA and the EC50 value was ~ 1.2 nM (**Figure 5E**). The purified monoclonal antibody recognized N protein in cell lysates of HCoV-OC43-infected Vero cells but not in MERS-CoV- or SARS-CoV-2-infected Vero cells (**Figure 5F**). Through ELISA analysis, we proved that the antibody specifically recognized HCoV-OC43 N protein-Bio-His_6_ but not MERS-CoV N protein-Bio-His_6_ or SARS-CoV-2 N protein-Bio-His_6_ (**Figure 5G**). Next, we confirmed a direct interaction between Spike CD and N protein in HCoV-OC43-infected cells. We incubated the purified HCoV-OC43 Spike CD-Fc or Fc control with lysates of HCoV-OC43 virus, pulled down complexes with Protein A agarose beads, and detected HCoV-OC43 N protein using the HCoV-OC43 N protein-specific monoclonal antibody (1C7D7 mAb). The results revealed that N protein of HCoV-OC43 specifically interacts with HCoV-OC43 Spike CD-Fc but not with the Fc control (**Figure 6**). The Fc control and HCoV-OC43 Spike CD-Fc recombinant proteins showed two protein bands composed of major monomers and minor dimers as we have seen in **Figure 4B** (**Figure 6B**).

### Detection of HCoV-OC43 by an ELISA System Based on the Interaction Between Recombinant Spike CD and N Proteins

**Figure 7:**
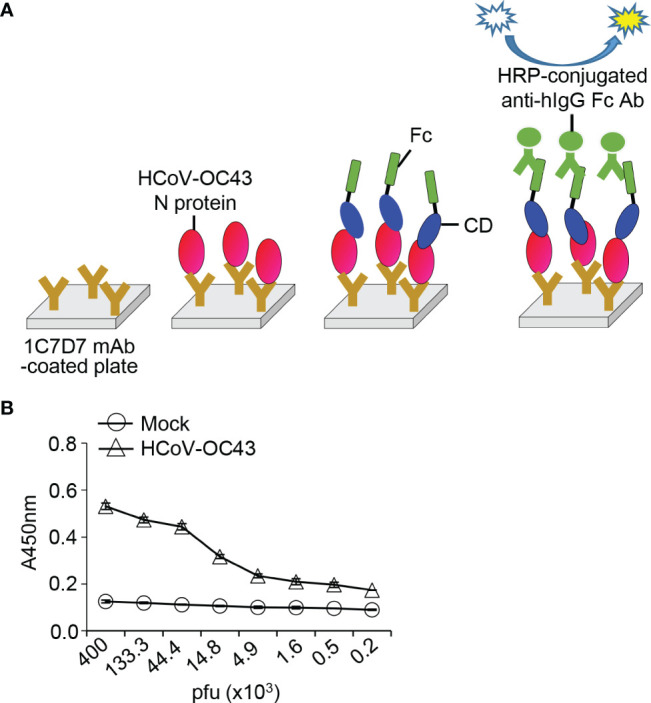
Detection of HCoV-OC43 in cell culture media with HCoV-OC43 N protein-specific monoclonal antibody and recombinant HCoV-OC43 Spike CD-Fc protein. **(A)** Schematic of the ELISA system. **(B)** HCoV-OC43 in cell culture supernatants were lysed with cell lysis buffer and serially diluted in PBST. The virus lysates were added into 96-well immunoplates coated with HCoV-OC43 N protein-specific monoclonal antibody (clone 1C7D7 mAb) and incubated for 2 h at room temperature. After washing with PBST, recombinant Spike CD-Fc protein was added, and then HRP-conjugated anti-human IgG Fc antibody was added to each well. The amount of HCoV-OC43 N protein in each well was determined by ELISA.

Similar to our previously reported “bait and prey” system (35), we designed an ELISA-based detection system for HCoV-OC43 using the anti-HCoV-OC43 N protein antibody and HCoV-OC43 Spike CD-Fc (**Figure 7A**). The HCoV-OC43 N protein-specific monoclonal antibody (1C7D7 mAb) was used to capture N proteins of HCoV-OC43 particles in lysates, and the N protein was allowed to interact with HCoV-OC43 Spike CD-Fc. This ELISA system successfully detected HCoV-OC43 in a concentration-dependent manner (**Figure 7B**), suggesting that the interaction between N protein and Spike CD Fc fusion protein can be applied as a novel method for the detection of HCoV-OC43

## Discussion

Since the emergence of SARS-CoV in 2002, research on highly pathogenic coronaviruses has heavily focused on identifying pathophysiological mechanisms as targets for novel therapeutics. For example, vaccines that suppress the infection mechanism of SARS-CoV-2 are now being administered worldwide, and many studies are being conducted to develop therapeutic agents for SARS-CoV-2. However, studies on other HCoVs, like HCoV-229E, HCoV-NL63, HCoV-HKU1, and HCoV-OC43, have yet to identify many therapeutic targets. Identifying targets for these and other coronaviruses is important because they not only cause most human common colds, but they also have the potential to cross species barriers with risky variants. Previously, we reported interaction of S and N proteins in MERS-CoV- and SARS-CoV-2-infected cells for the assembly of the viruses. Furthermore, we verified that the Spike CDs of MERS-CoV and SARS-CoV-2 are involved in the interaction with the N protein of each corona virus. In addition, we suggested that interactions of S and N protein are required for packaging of the viral genome as reduction of interactions between S and N protein by cell-penetrating Spike CD of MERS-CoV and SARS-CoV-2 treatment resulted in reduced viral protein expression and virus replication (25). Here, we investigated the interaction of HCoV-OC43 Spike CD and N protein with the hope of identifying a new therapeutic target for HCoV-OC43. We confirmed that Spike CD and N protein interact in HCoV-OC43 and found that the cell-penetrating Spike CD HCoV-OC43 peptide inhibited the production of HCoV-OC43 in infected cells. Taken together, our results indicate that the interaction of Spike CD and N protein is conserved across coronavirus and these interactions play an important role in coronavirus replication. Therefore, this would be a fantastic therapeutic target for future coronavirus treatments.

To detect interaction between SARS-CoV-2 Spike CD and N protein, we recently developed a novel assay based on the bait and prey ELISA system using the recombinant fusion proteins SARS-CoV-2 Spike CD-Fc and SARS-CoV-2 N-Bio-His_6_ (35). Here, we applied the same strategy to HCoV-OC43. We expressed the HCoV-OC43 Spike CD-Fc and HCoV-OC43 N-Bio-His_6_ recombinant proteins and confirmed specific and direct interaction between these proteins *in vitro*. Furthermore, we confirmed that the system can be modified to detect HCoV-OC43 using anti-HCoV-OC43 N protein-specific monoclonal antibody that we produced in this study. Taken together, our data suggest that the Spike CD-N protein interaction may be conserved among coronaviruses and this interaction can be used as a quantitative measurement of respective virus particles using our bait and prey ELISA system. Moreover, we believe that our ELISA system can be used to screen for potential drugs that inhibit the Spike CD-N protein interaction and thereby block coronavirus replication.

## Data Availability Statement

The raw data supporting the conclusions of this article will be made available by the authors, without undue reservation.

## Ethics Statement

The animal study was reviewed and approved by Hallym University Institutional Animal Care and Use Committee.

## Author Contributions

H-JK and YL conceived of the project. H-JK and YL designed the experiments and wrote the manuscript. JK, MYK, DK, SP, MJK, KB, J-KC, SM, and MA carried out the experiments. H-JK, JK, MYK, DK, and YL analyzed the data. All authors contributed to the article and approved the submitted version.

## Funding

This research was supported by grants from the National Research Foundation (NRF-2019M3A9E4032628, NRF-2020M3A9I2107294) funded by the Ministry of Science and ICT in the Republic of Korea.

## Conflict of Interest

The authors declare that the research was conducted in the absence of any commercial or financial relationships that could be construed as a potential conflict of interest.

## Publisher’s Note

All claims expressed in this article are solely those of the authors and do not necessarily represent those of their affiliated organizations, or those of the publisher, the editors and the reviewers. Any product that may be evaluated in this article, or claim that may be made by its manufacturer, is not guaranteed or endorsed by the publisher.
